# Use of Saliva for Early Dengue Diagnosis

**DOI:** 10.1371/journal.pntd.0001046

**Published:** 2011-05-10

**Authors:** Grace Yap, Bijon Kumar Sil, Lee-Ching Ng

**Affiliations:** Environmental Health Institute, National Environment Agency, Singapore, Singapore; Universidade de São Paulo, Brazil

## Abstract

**Background:**

The necessity of a venous blood collection in all dengue diagnostic assays and the high cost of tests that are available for testing during the viraemic period hinder early detection of dengue cases and thus could delay cluster management. This study reports the utility of saliva in an assay that detects dengue virus (DENV)–specific immunoglobulin A (Ig A) early in the phase of a dengue infection.

**Methods and Findings:**

Using an antigen capture anti-DENV IgA (ACA) ELISA technique, we tested saliva samples collected from dengue-confirmed patients. The sensitivity within 3 days from fever onset was over 36% in primary dengue infections. The performance is markedly better in secondary infections, with 100% sensitivity reported in saliva samples from day 1 after fever onset. Serum and salivary IgA levels showed good correlation (Pearson's *r* = 0.69, *p*<0.001). Specificity was found to be 97%.

**Conclusion:**

Our findings suggest that this technique would be very useful in dengue endemic regions, where the majority of dengue cases are secondary. The ACA-ELISA is easy to perform, cost effective, and especially useful in laboratories without sophisticated equipment. Our findings established the usefulness and reliability of saliva for early dengue diagnosis.

## Author Summary

The importance of laboratory diagnosis of dengue cannot be undermined. In recent years, many dengue diagnostic tools have become available for various stages of the disease, but the one limitation is that they require blood as a specimen for testing. In many incidences, phlebotomy in needle-phobic febrile individuals, especially children, can be challenging, and the tendency to forgo a dengue blood test is high. To circumvent this, we decided to work toward a saliva-based assay (antigen-capture anti-DENV IgA ELISA, ACA-ELISA) that has the necessary sensitivity and specificity to detect dengue early. Overall sensitivity of the ACA-ELISA, when tested on saliva collected from dengue-confirmed patients (EDEN study) at three time points, was 70% in the first 3 days after fever onset and 93% between 4 to 8 days after fever onset. In patients with secondary dengue infections, salivary IgA was detected on the first day of fever onset in all the dengue confirmed patients. This demonstrates the utility of saliva in the ACA-ELISA for early dengue diagnostics. This technique is easy to perform, cost effective, and is especially useful in dengue endemic countries.

## Introduction

Dengue is one of the most prevalent mosquito-borne diseases in humans. This disease is best controlled by regular *Aedes* source reduction activities. However, total eradication of *Aedes* in a densely populated urban area where the vector has established itself is a daunting task. Dengue control must include prompt control response to dengue clusters, and early and reliable diagnosis of cases is critical to this effort, which aims to halt the DENV transmission. There has been progress in recent years in the development of dengue diagnostic tools, resulting in the availability of suitable tests for each stage of the disease.

Specific detection of dengue viral ribonucleic acid (RNA) using real-time reverse transcription (RT) polymerase chain reaction (PCR) is widely utilized to diagnose and serotype dengue infections in the early phase of the disease [1]–[7]. These techniques, while rapid and effective in providing early dengue diagnosis, are costly and require trained personnel to perform. It is thus only currently available in a limited number of clinical laboratories. The more recent development of DENV non-structural protein 1 (NS1) antigen detection in the Enzyme-linked immunosorbant assay (ELISA) and rapid lateral flow platform has offered clinical laboratories an effective tool for early diagnosis during the febrile phase of the disease [8]–[13].

The detection of anti-DENV immunoglobulin M (IgM) is the most widely used serological assay in dengue diagnosis [14]–[21]. However, anti-DENV IgM is usually detected 5 to 6 d after the onset of fever and thus could result in a delay in diagnosis. Moreover, it can persist for more than 8 mo [20], [22], [23], and in dengue-endemic countries such as Singapore, the detection of IgM in a febrile patient does not necessarily indicate an acute dengue infection. The requirement for analysis of paired samples collected at least 7 d apart, for definitive diagnosis, could delay intervention efforts.

Unfortunately, the necessity of a venous blood collection in all available dengue diagnostic assays and the high cost of the tests that are available for the viraemic period hinder the early detection of cases and clusters. Phlebotomy in needlephobic febrile individuals, especially children, can be challenging, and the tendency to forgo a dengue blood test is high.

We have therefore worked toward saliva-based techniques that could address the early phase of the disease. Saliva is known to be rich in IgA, the concentration of which is 100 times greater than that of IgM and 14 times greater than IgG, and should thus serve as a good target for early diagnosis [24]. Usage of salivary IgG for diagnosis and epidemiological studies has been described before [24]–[26]. The use of serum anti-DENV IgA as a diagnostic marker has previously been explored. Groen et al. [27] described the simultaneous increase of DENV-specific IgA and IgM in dengue patients and reported that IgA was short-lived compared to IgM [27]. An antibody-capture IgA (AAC) ELISA was used. Using the same technique, subsequent studies showed that anti-DENV IgA typically appeared after IgM did and was thus not suitable for dengue diagnostics [15], [23], [28]. The use of salivary IgA for disease detection has also been reported for Human Immunodeficiency Virus, Hepatitis A and B, Measles, Mumps, and Rubella [29]–[33].

In this prospective study, we developed a protocol that allows saliva to be used for anti-DENV IgA detection. The technique, antigen-capture anti-DENV IgA (ACA)-ELISA, not only increased the sensitivity of DENV-specific IgA detection, it also reduced the total test time to 90 min, when compared with a previously published IgA assay.

## Materials and Methods

### Samples for Detection of Anti-DENV IgA

The Environmental Health Institute (EHI) is a national public health laboratory that functions as a licensed diagnostic laboratory, with an ISO9001 accreditation, as well as a research laboratory.

Three suites of characterized samples, collected in Singapore, were used in this study. WHO criteria for dengue confirmation was adhered to for the determination of dengue status in the following samples: The first (A) comprised saliva and sera collected from 10 healthy volunteers as well as dengue-confirmed patients for optimization of the protocol. The sera from healthy volunteers were previously confirmed to be dengue negative via DENV RT-PCR and PanBio IgM Capture ELISA, and their negative anti-DENV IgA status was ascertained in both saliva and sera using a previously reported DENV Antibody Capture IgA ELISA (AAC-ELISA) [23]. The samples from dengue-confirmed patients consisted of saliva and sera sequentially collected from five patients during the acute phase of their disease. The dengue status of these patients was confirmed by RT-PCR on sera collected in the first 72 h and subsequent sero-conversion as demonstrated by IgM assays. These five sets of saliva and sera samples were also previously confirmed to be DENV IgA positive in AAC-ELISA. Samples in this suite were used as reference samples to establish the ACA-ELISA.

The second suite of samples (B), for evaluation of the newly developed ACA-ELISA protocol, consisted of saliva and sera obtained from 69 DENV-PCR-confirmed patients through three consecutive collections. The first collection was within 72 h after fever onset, second collection around 3 d after first collection, and third collection within 21 d after fever onset. IgM tests, performed on all three collections of each patient, demonstrated sero-conversion of each of the patients, thus confirming their dengue status. Of the 69 patients, 37 (53.3%) had DENV1 infections, 4 (5.8%) had DENV2, and 28 (40.6%) had DENV3, as determined by RT-PCR on the first collection samples [34]. Of the 69 PCR-positive patients from suite B samples, there were 33 primary infections and 36 secondary infections. A primary dengue infection was characterized by first collection serum (first 72 h) being positive for DENV PCR but PanBio Indirect IgG ELISA negative. The absence of DENV-specific IgG in the acute phase of a dengue infection is indicative of primary dengue infection [35]–[37]. A secondary dengue infection was characterized by the first collection sample being concurrently positive for DENV PCR and IgG. These samples were collected through a research project (EDEN) from April 2005 to December 2006 [34] and were used for evaluation of the established ACA-ELISA protocol.

The third suite (C), serving as a specificity test of the ACA-ELISA, comprised three consecutive collections of saliva and sera from 75 DENV PCR-negative febrile patients (EDEN). Collected in the same manner as suite B, they were DENV RT-PCR negative patients, and IgM tests revealed no sero-conversion among all 75 cases.

### Saliva Collection

Oracol saliva collection swab (Malvern Medical Development Lid, UK) was used for saliva collection. A standardized saliva collection protocol was used such that active saliva secretion was obtained. Patients were instructed to swab in a scrubbing manner their inner upper and lower cheeks 10 times each on both sides and place the swab under their tongues for 1 min. Saliva samples, together with blood, were transported on ice, processed within the same day of collection, and stored at −80°C until testing.

### Ethics Statement

Use of samples in suite A was approved by NEA's Bioethics Committee (IRB004.1). Use of samples in suites B and C was approved by the National Healthcare Group Internal Review Board (DSRB B/05/013). Written informed consent was obtained from all participants.

### Dengue Antigen

DENV cell culture lysate antigens used in ACA-ELISA were prepared using a DENV 2 strain (SS194Y02) of the Cosmopolitan genotype, isolated from a dengue patient locally, according to the method previously described [38], and viral titre was determined via plague assay [39]. A single batch of cell lysate was prepared and utilized for this entire study.

### ACA-ELISA

Checkerboard dilution was performed using anti-DENV IgA positive and negative samples from suite A as reference to optimize the ACA-ELISA for serum and saliva usage. In brief, the 96-well plate maxisorp plates (Nunc, Denmark) were coated with 100 µl/well of pan-DENV monoclonal antibodies (Mab; 1.15 mg/ml; Immunology Consultants Laboratory, USA) diluted at 1∶500 in sodium bicarbonate (pH 9.5) and incubated either overnight at 4°C or 1 h at 37°C. After blocking the plate with dilutent buffer (5% skim milk containing 0.05% Tween-20), virus lysate (2.14×10^6^ pfu/ml) in diluent buffer was added to each well and incubated at 37°C for 1 h. The plate was then washed six times using washing buffer (1X PBS-0.05% Tween-20). Either 100 µl of test serum at 1∶100 in diluent buffer or 100 µl of saliva at 1∶5 dilution was added to each well. In each plate, two positive controls, two negative controls (DENV negative human sera or saliva), and one plate control (no sera or saliva added) were included. The two positive controls were either two anti-DENV IgA positive sera (titre of 1∶256) or two anti-DENV IgA positive saliva samples (titre 1∶10). After 1 h of incubation at 37°C, the plate was washed again six times, and 100 µl of 1∶4000 rabbit anti-human IgA conjugated with horse-radish peroxidase (HRP; Dako, Denmark) was added to each well and then incubated for 30 min at 37°C. Following incubation, the plate was then washed again six times, and 100 µl of tetra-methyl-bencidine (TMB; Sigma, USA) was added to each well and incubated for 5 min at room temperature. Further color development was stopped using 100 µl 0.5 M sulphuric acid, and absorbance was measured at 450 nm against a reference filter at 620 nm. The same batch of controls, reagents, and dengue lysate was used for this entire study. All IgA assays for evaluation of the protocol were performed by a single analyst within a day, without masking the results of the reference tests. Interval between sample collection and testing ranged from 1 mo to 1 y, during which samples were kept frozen at −80°C.

### PCR, IgM, and IgG Assays

All IgM and IgG assays were performed using PanBio IgM Capture ELISA and PanBio Indirect IgG ELISA. IgM, IgG assays, and RT-PCR (7) were conducted by five trained personnel of the EHI Diagnostics Unit, licensed by the Ministry of Health. While PCR tests were performed within a day of collection of samples, antibody tests on sera and saliva were performed either on the same day or within 1 mo, during which samples were frozen at −80°C.

### Data Analysis

Data analysis, including calculations of correlation coefficients and standard error of proportion, was carried out using Microsoft Excel 2002 and Statistical Package for Social Sciences (SPSS) 17.0. Sensitivity and its 95% confidence intervals were provided as estimates of the effectiveness of the in-house ACA-ELISA. Standard error of proportion was calculated using the formula √ [p(1−p)/n].

## Results

### Cutoff Point for IgA Assay

The cutoff point of saliva IgA assays was determined with suite A of saliva samples from healthy volunteers. Mean ± standard deviation optical density (OD) values of the negative controls were determined. A sample was considered negative when the OD value was less than the mean value for the negative control plus two standard deviations, equivocal when the OD value exceeded the mean value for the negative control plus 2 standard deviations but less than 3 standard deviations and considered positive when the OD value is above 3 standard deviations.

### ACA ELISA and IgM ELISA on Saliva Samples

**Figure 1:**
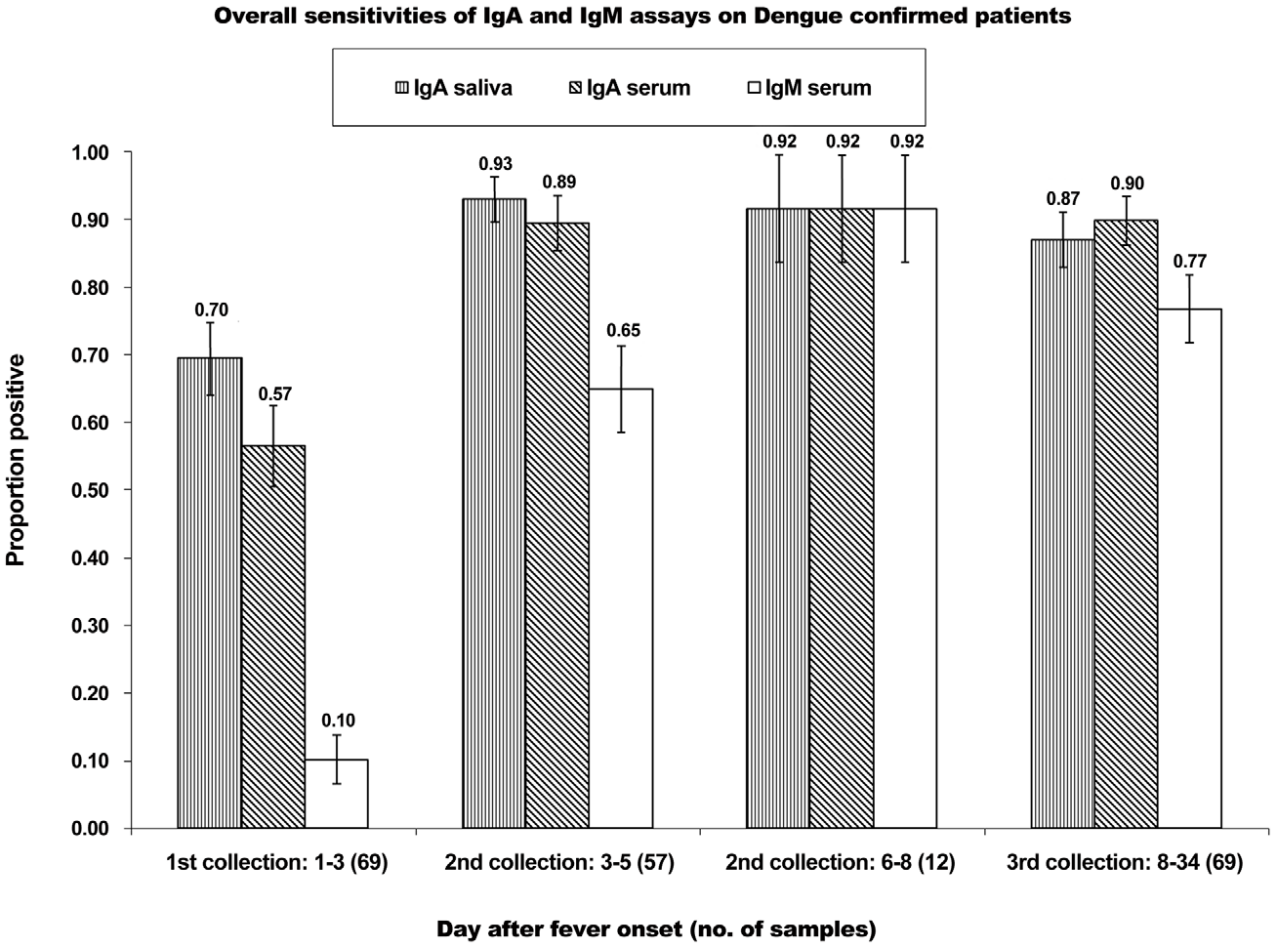
Sensitivity of ACA-ELISA assay compared with Capture IgM ELISA. Overall sensitivity of ACA-ELISA in saliva and sera and Capture-IgM ELISA in sera indicated the reliability of ACA-ELISA (saliva) in picking up 7 times more DENV patients compared to Capture-IgM ELISA (sera) in the first 3 d after fever onset. The error bars showed 1 standard of deviation of the proportion for each value.

Suite B saliva and serum samples, consisting of three consecutive collections from each dengue-confirmed patient, were assayed for anti-DENV IgA using ACA-ELISA. All the serum samples were also tested with PanBio Capture IgM ELISA. Serum and salivary anti-DENV IgA levels showed good correlation (Pearson's *r* = 0.69; *p*<0.001). Figure 1 shows the sensitivities of ACA-ELISA on saliva and sera, compared to IgM Capture ELISA on sera. ACA-ELISA on saliva had an overall sensitivity of 70% in the first 3 d after fever onset and subsequently rose to over 90% between 3 and 8 d after fever onset. The same technique on sera gave similar results. More interestingly, the sensitivity of ACA-ELISA in saliva was higher than that of IgM Capture ELISA on sera, which detected only 10% of the dengue-confirmed patients after 1 to 3 d of fever, and only rose to around 90% after day 6 of fever. The specificity of the ACA-ELISA test was also found to be high at 97%. Among the 75 DENV-negative patients in suite C, only one patient tested positive, at day 7 and 27, respectively.

### ACA-ELISA in Primary and Secondary Dengue Infection

**Figure 2:**
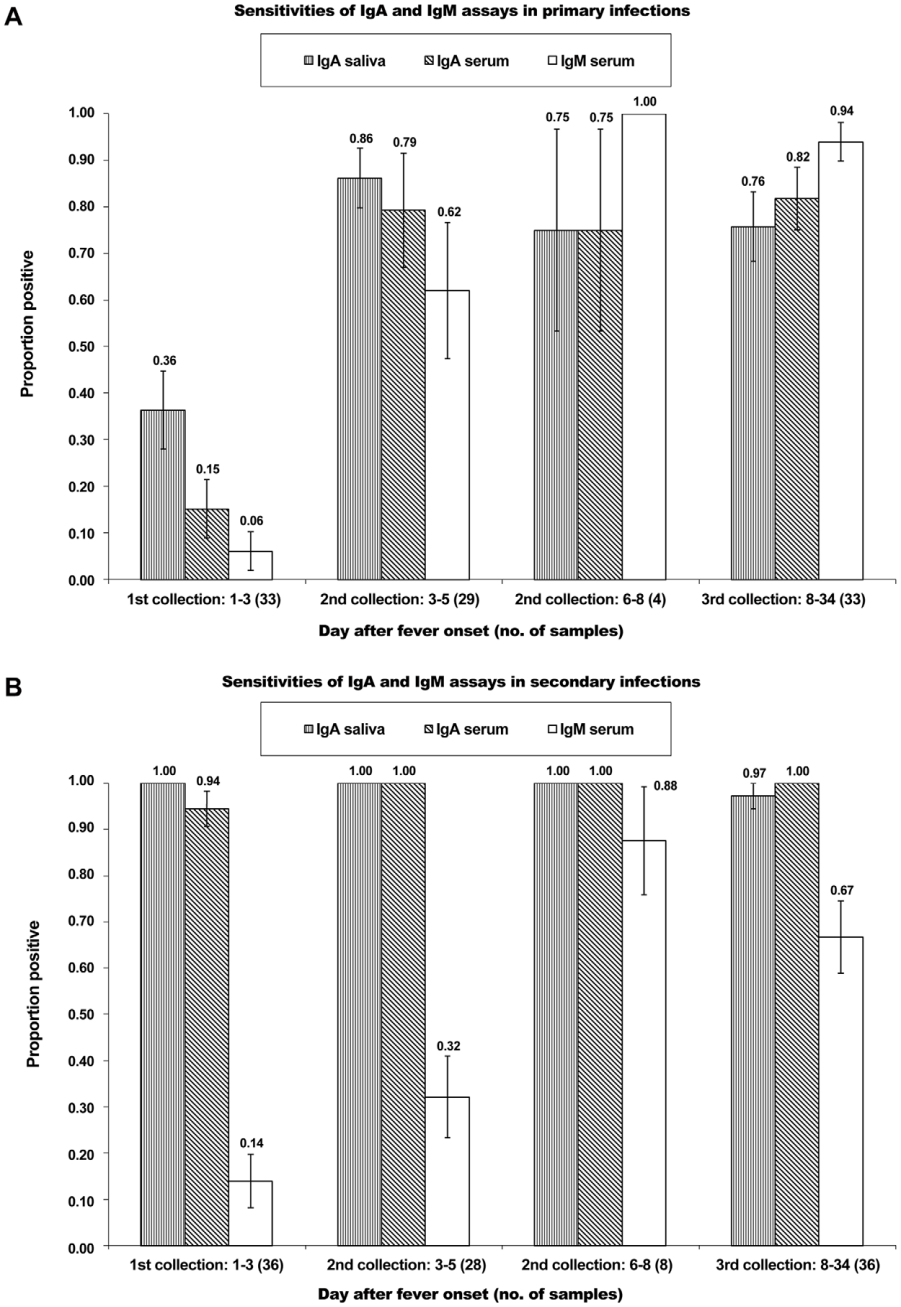
Sensitivity of ACA-ELISA and Capture-IgM ELISA in primary and secondary dengue. Data showed the excellent sensitivity of ACA-ELISA among secondary cases. The ACA-ELISA was less sensitive in primary DENV cases (A), when compared to secondary cases (B), but was notably more sensitive than IgM Capture ELISA. The error bars showed 1 standard of deviation of the proportion for each value.

The data of the ACA-ELISA were further analyzed with respect to primary (*n* = 33) and secondary (*n* = 36) infections. The sensitivity of the technique is detailed in Figure 2A (primary infection) and 2B (secondary infection). Among the 33 primary cases, the sensitivity of ACA-ELISA on saliva was 36% in the first 3 d and rose to 86% in the second collection (3 to 5 d). The number of samples collected during 6 to 8 d was small. Nevertheless, the results showed that at the early phase of the disease, the sensitivity of ACA-ELISA on saliva was clearly higher than those of ACA-ELISA and IgM Capture on sera (15% and 6% in the first 3 d after fever onset, respectively). Interestingly, ACA-ELISA tested on the saliva and sera of 36 secondary cases yielded sensitivities of 100% and 94%, respectively, in the first 3 d of fever and continued to allow detection at this high rate in second and third collection (3 to 34 d). In contrast, IgM Capture ELISA on sera of secondary cases gave a detection sensitivity of 14% in the first 3 d and rose to 88% only by day 6.

## Discussion

This study has demonstrated the potential use of saliva for early dengue diagnostics. The sensitivity of ACA-ELISA was 70% to 92% within the first 8 d from onset of fever. Data from our diagnostic unit have revealed that dengue patients in local settings visit the primary health care physicians at an average of 3.5 d from onset of fever [13]. Ninety-five percent of the patients would visit their doctors within 0.95 to 6.03 d after fever onset. Under this setting, we have found that our in-house RT-PCR offers 72.5% specificity and the most sensitive commercial NS1 assay offers 81.7% [40]. Therefore, ACA-ELISA on saliva is comparable to these early diagnosis tests and offers the additional advantages of non-intrusive sampling and easy cost-effective laboratory procedures.

It is not surprising that among the secondary dengue infections, IgA was detected in both the saliva and sera of all individuals as early as day 1 after fever onset, while detection in primary cases was delayed. This is due to the presence of IgA memory cells from the previous dengue infection that was triggered by the secondary DENV exposure—not unlike the early IgG responses in secondary dengue cases. This has also been observed in previous studies [15], [25], [41], [42]. Due to the difference in sensitivity between primary and secondary cases, the sensitivity of the ACA-ELISA in a population will be highly dependent on the proportion of secondary cases in the cohort. About 50% of all dengue cases in Singapore are secondary [34]. Under this circumstance, we demonstrated about 70% sensitivity in the first 3 d of fever. In a population with a higher rate of secondary cases, the sensitivity could potentially be higher.

There are two possible explanations to the early detection of IgA even in primary cases. Firstly, inaccuracy in reporting of the onset dates, due to insensitivity to mild fever or bias in recalls, may contribute to the situation. Secondly, intrinsic incubation periods and response time vary among individuals. Dengue patients are infected 2 to 14 d before fever onset, a period which may have allowed IgA (and IgM) production in some individuals. Early detection of IgM for some individuals is also evident for dengue and chikungunya [43], [44]. It is highly likely that in the very early phases of dengue, IgM and IgA are present in very low levels, and time of detection lies in the sensitivity of the technique used.

Previous studies, using AAC-ELISA, reported low sensitivities when using salivary IgA, in contrast to our findings [15], [24], [25], [45]. Two strategies were designed in this study to overcome the limitation seen in previous studies. Firstly, saliva collection protocol in this study was designed to allow for the collection of actively secreted saliva. Previous studies on DENV-specific IgA in saliva used passively secreted saliva in their evaluation. Prior to this study, a comparison of the two saliva collection protocols had revealed that, in the same individuals, actively collected saliva yielded higher levels of DENV-specific IgA than passively collected ones (unpublished data, Yap G). Secondly, ACA-ELISA was designed to eliminate binding competition from high levels of non-specific IgA that are normally present in mucosal secretions to protect one from infectious diseases. A previously published technique, AAC-ELISA, captured all IgA in the first step, followed by subsequent differentiation of DENV-specific IgA from the pool of IgAs. In the event of a dengue infection, anti-DENV IgA may represent only a small proportion of IgA in saliva. The low detection rate of anti-DENV IgA could be due to preexisting non-DENV-specific IgA out-competing anti-DENV IgA. To circumvent the limitation, ACA-ELISA was designed to capture all anti-DENV antibodies in its first step, followed by detection of anti-DENV IgA. In the early phase of a primary dengue infection, DENV-specific IgM and IgG are present in low levels, and coupled with the enrichment of IgA in saliva, the approach is expected to increase the detection sensitivity. The high sensitivity of the technique could also explain the observations that in the early phases of the disease in primary cases, DENV IgA could be detected earlier than DENV IgM, appearing to go against classical immunology. The capture IgM ELISA used in this study, like the AAC-ELISA, was expected to pick up only elevated levels of DENV IgM. The difference thus lies on the sensitivity of the techniques used. The slightly higher sensitivity in saliva compared to that in serum is likely due to the dimeric structure of the secretory IgA, which could increase the amplification of signal output from the ELISA.

Even though the antigen used in ACA-ELISA of this study was DENV2 and not a tetravalent antigen, the ACA-ELISA is effective in detecting IgA illicited by the three serotypes circulating in Singapore during the study period, as DENV IgA, like IgM, is cross-reactive with all four serotypes [46]. This is supported by a study that demonstrated no significant differences in the sensitivity of ACA-ELISA when DENV2 is replaced by a tetravlent antigen (unpublished data, Yap G). A multi-country study is ongoing to evaluate the test in various epidemiology settings, particularly to establish its performance in settings with other circulating DENV genotypes and other diseases that illicit cross-reacting antibodies, which may impact its performance. Potential limitation in specificity can be circumvented using recombinant antigen specific to DENV.

This study suggests the potential of the saliva ACA-ELISA for dengue diagnosis. It eliminates the need to collect blood from dengue-suspected patients, is painless, is non-intrusive, and reduces the risk of needle stick injury. Moreover, the ELISA-based technique is simple and cost effective. Patients, especially the very young and the old, will be more willing to undergo a dengue test. Together, these benefits can potentially improve surveillance and early detection of cases, which in turn can translate to prompt dengue control effort. Due to its high sensitivity among secondary dengue infections, this technique could be very useful in highly endemic areas where the majority of the dengue cases are secondary.

## Supporting Information

Checklist S1 STARD Checklist, Diagnostics Checklist.
(0.05 MB DOC)

Figure S1 Flow chart for evaluation of Dengue Saliva IgA Test.
(0.04 MB DOC)
