# Supplementary material for: Use of Saliva for Early Dengue Diagnosis
Source: PLoS Negl Trop Dis. 2011 May 10;5(5):e1046. doi: 10.1371/journal.pntd.0001046 (PMC3091836; doi:10.1371/journal.pntd.0001046)
Supplement: Figure S1 — Flow chart for evaluation of Dengue Saliva IgA Test. (0.04 MB DOC) [file pntd.0001046.s002.doc]

# Supporting Figure S1 - Flow chart for evaluation of Dengue Saliva IgA Test

Patients with fever not more than 72 hours

Enrolment and first collection of sera and saliva, n=184

Excluded due to incomplete collection of consecutive samples, n=40

n= 40

Consecutive samples of sera and saliva collected. 2^nd^: around three days after first collection; 3rd within 21 days after fever onset.

## DENV RT-PCR +ve on first samples

Suite B, n=69 patients

## DENV RT-PCR -ve on first samples

Suite B, n=75 patients

Serotype determination with first samples

IgG and IgM assays of all three collections of sera to confirm dengue status

Sero-conversion demonstrated by all patients, n=69 patients

No sero-conversion demonstrated by all patients, n=75 patients

Secondary infection as demonstrated by IgG +ve in Day1-3 samples

Secondary cases: 36 patients

Primary cases: 33 patients

ACA-ELISA (IgA) Sensitivity test

ACA-ELISA (IgA) Specificity test

IgA +ve:

Day1-3, n=36

Day 3-5, n=36

IgA -ve:

Day1-3, n=0

Day 3-5, n=0

IgA +ve:

Day1-3, n=12

Day 3-5, n=28

IgA -ve:

Day1-3, n=21

Day 3-5, n=5

IgA +ve:

n=1

IgA -ve:

n=74

ACA-ELISA on all 3 collections
